# Peripheral neuropathy in a diabetic child treated with linezolid for multidrug-resistant tuberculosis: a case report and review of the literature

**DOI:** 10.1186/s12879-017-2499-1

**Published:** 2017-06-12

**Authors:** Aravind Swaminathan, Philipp du Cros, James A. Seddon, Shamsiya Mirgayosieva, Rajabov Asladdin, Zulfiya Dusmatova

**Affiliations:** 1 Médecins Sans Frontières (MSF), Dushanbe, Tajikistan; 2 Médecins Sans Frontières (MSF), Manson Unit, London, UK; 3 Imperial College London, London, UK; 4 National Tuberculosis Programme, Ministry of Health and Social Protection of the Republic of Tajikistan, Dushanbe, Tajikistan; 5 Department of Paediatrics, Saveetha Medical College and Hospital, Thandalam, Kancheepuram, Tamil Nadu India

**Keywords:** Case report, Multi drug resistant tuberculosis, Linezolid, Neuropathy, Diabetes mellitus

## Abstract

**Background:**

Extensively drug-resistant (XDR) tuberculosis (TB) and multidrug resistant (MDR)-TB with additional resistance to injectable agents or fluoroquinolones are challenging to treat due to lack of available, effective drugs. Linezolid is one of the few drugs that has shown promise in treating these conditions. Long-term linezolid use is associated with toxicities such as peripheral and optic neuropathies. Diabetes mellitus (DM), especially when uncontrolled, can also result in peripheral neuropathy. The global burden of DM is increasing, and DM has been associated with a three-fold increased risk of developing TB disease. TB and DM can be a challenging combination to treat. DM can inhibit the host immune response to tuberculosis infection; and TB and some anti-TB drugs can worsen glycaemic control. A child experiencing neuropathy that is a possible complication of both DM and linezolid used to treat TB has not been reported previously. We report peripheral neuropathy in a 15-year-old boy with type 1 DM, diagnosed with MDR-TB and additional resistance to injectable TB medications.

**Case presentation:**

The boy was treated with a linezolid-based regimen, but after 8 months developed peripheral neuropathy. It was unclear whether the neuropathy was caused by the DM or the linezolid therapy. He had clinical improvement following cessation of linezolid and was declared cured following 21 months of treatment. Following completion of treatment, nerve conduction studies demonstrated significant improvement in neuropathy.

**Conclusions:**

To the best of our knowledge, this is the first case of peripheral neuropathy reported in a diabetic child on long-term linezolid therapy for tuberculosis. This case study underlines the importance of stringent follow-up for side effects of linezolid, especially when associated with co-morbidity such as DM that increases the chances of adverse effects. The presence of both DM and TB should alert a physician to strive for optimal glycaemic control to minimize the risk of complications as well as optimizing the chances of recovery from TB. Our case report shows the need for close and frequent monitoring for neuropathy to enable early intervention and thereby a favourable outcome in children who may otherwise suffer a long-lasting, debilitating, and painful neuropathy.

## Background

Multidrug-resistant (MDR) tuberculosis (TB) is defined as TB with resistance to isoniazid and rifampicin, while extensively drug-resistant (XDR)-TB is defined as MDR-TB with additional resistance to at least one injectable agent and a fluoroquinolone. Anti-TB drugs of unclear efficacy (categorised as group 5) can be used to construct treatment regimens in children with advanced drug resistance. In this group, the oxazolidinone linezolid appears to be effective. However, linezolid is associated with considerable adverse effects, especially when used at a high dosage for a long duration. The recent World Health Organisation MDR-TB guidelines moving linezolid to group C will likely lead to greater use of Linezolid in MDR-TB treatment, and with increasing DM co-morbidity the management of peripheral neuropathy in this patient group is likely to occur more frequently [1].

The management of diabetes mellitus (DM) requires a combination of lifestyle interventions, dietary modifications, and anti-diabetic medications, with subcutaneous injections of insulin required for type 1 DM. Glycaemic control can be challenging to sustain in children, and more so when DM is associated with co-morbidities (such as TB) that may further hamper control. In addition, inflammation associated with active tuberculosis can cause insulin resistance [2]. As a result, children are at risk of complications such as neuropathy.

Here we present a case of a child with type 1 DM, diagnosed with MDR-TB and additional resistance to injectable TB medications, who developed peripheral neuropathy after 8 months of treatment with a linezolid-based regimen.

## Case presentation

**Fig. 1:**
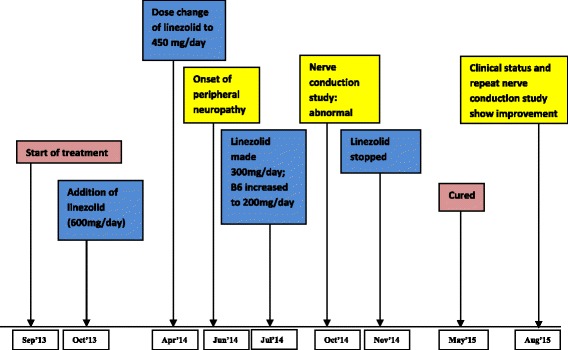
Timeline of events. The figure represents the timeline of events in the described patient starting with beginning of treatment, occurrence of neuropathy, intervention and subsequent improvement in follow up

A 15-year-old boy, weighing 50 kg, presented to an urban clinic in Dushanbe, Tajikistan, with clinical features of TB disease. He had been diagnosed with type 1 DM 5 years earlier, and had been treated with insulin. However, the monitoring of his blood sugar levels had been erratic and his glycaemic control had not been optimal. A sputum sample was culture-positive for *Mycobacterium tuberculosis*, with a drug susceptibility test (DST) profile demonstrating resistance to isoniazid, rifampicin, streptomycin, kanamycin, capreomycin, and amikacin and susceptibility to pyrazinamide, ethambutol, and ofloxacin. He was started on a regimen of pyrazinamide, ethambutol, capreomycin, moxifloxacin, prothionamide, cycloserine, linezolid, amoxicillin/clavulanate, and clofazimine. Linezolid was initially started at a dose of 600 mg once daily (Fig. 1). Treatment included initial hospitalisation with attention to blood sugar management, and after discharge, logistical support for sugar monitoring and regular endocrinologist follow up were instituted.

After 6 months, the boy showed a good clinical and bacteriological response and, in light of the risk of adverse effects, the dose of linezolid was reduced to 450 mg once daily. During the eighth month of treatment he developed peripheral neuropathy (paraesthesia and mild intermittent pain) in both feet. There was no objective clinical evidence of cranial or peripheral nerve palsies; sensory perception, muscle tone, power, and deep tendon reflexes were normal. Ishihara tests performed each month did not show any abnormalities indicative of optic neuritis. Detailed examination by an ophthalmologist was normal. Diabetes control was optimized with increasing insulin requirements. The dose of linezolid was further reduced to 300 mg once daily and pyridoxine increased from 150 mg to 200 mg once daily. Although there was transient improvement in pain, nerve conduction and electromyography studies demonstrated sensory and motor peripheral nerve damage in both lower limbs with more severe involvement of the right-sided motor components. Colour duplex ultrasonography of the lower limb arteries did not reveal any abnormalities. Linezolid was stopped with the rest of the regimen continuing.

The boy showed clinical improvement following cessation of linezolid and was declared cured following a total of 21 months of treatment. Nerve conduction studies were repeated 10 months after the initial evaluation. A significant improvement in motor nerve function on the right side was seen. The amplitude of M response improved between the two evaluations: markedly in right tibial nerve from 0.12 to 6.38 mV (normal: 13 mV); and marginally in right peroneal nerve from 0.1 to 0.3 mV (normal: 12 mV). The area of M response also improved: right tibial nerve from 1.2 to 18.2 mV ms (normal: 34.8 mV ms); and right peroneal nerve 1.2 to 4.2 mV ms (normal: 8.12 mV ms). Although sensory impairment was clinically less prominent than motor impairment, the amplitude of the action potential in the right sural nerve improved from 4 to 7 μV (normal: 11.4 μV). The boy also developed a transient thrombocytopenia after 6 months of therapy, which resolved prior to any dose reduction.

## Discussion

This case report demonstrates the development of peripheral neuropathy potentially caused by DM and/or linezolid treatment, and its resolution following early detection and appropriate management. The global burden of DM is increasing, and DM has been associated with a three-fold increased risk of developing tuberculosis disease [3]. With increasing use of linezolid for XDR-TB, the management of peripheral neuropathy in settings with a high prevalence of drug-resistant TB is likely to be a frequent occurrence. The improvement in motor and sensory neuropathy following cessation of linezolid in our patient is encouraging. Linezolid is emerging as an important anti-TB drug in the management of MDR-TB with or without associated resistance to injectable agents or fluoroquinolones.

Our patient had been treated for type 1 DM with insulin injections for 5 years, however, his follow-up had been irregular due to limited availability of an endocrinologist and a lack of reliable laboratory facilities close to his home. Although DM-related neuropathy is not usually seen until a number of decades following the diagnosis of DM, poor glycaemic control may have accelerated the contribution of DM to neuropathy in this boy. Several mechanisms of diabetic neuropathy have been postulated. Chronic hyperglycaemia [4] with lack of insulin and C-peptide seem to be triggers. This can lead to activation of the polyol pathway and protein kinase C, with increased advanced glycosylation end products and subsequent activation of transcription factor- κβ. This can lead in turn to pro-inflammatory gene expression, functional deficits of nitric oxide, and alterations in endothelial derived relaxing factor, which can result in microvascular reactivity and structural microangiopathy. These pathological processes culminate in axonal atrophy with progressive demyelination, and neuronal apoptosis with decreased fibre regeneration. It is also possible that endoplasmic reticulum stress might contribute to diabetic peripheral neuropathy [5].

A systematic review and meta analysis [6] that looked into 23 studies conducted in 14 countries comprising 507 patients reported a combined proportion of 29.92% for neuropathy in DRTB patients treated with linezolid. The incidence of neuropathy leading to permanent discontinuation of linezolid showed no significance upon dose comparisons (≤600 mg vs >600 mg).

A retrospective review [7] of 16 patients with MDR TB (including 10 XDR-TB) patients in New York revealed that seven of them developed neuropathy (peripheral and/or optic). Discontinuation of linezolid was required in three patients. While optic neuropathy resolved in one patient with discontinuation of linezolid, none of the patients with peripheral neuropathy had resolution despite stopping the drug.

A retrospective review [8] of seven children on linezolid-containing regimens for DR-TB in South Africa showed that neuropathy occurred in one child, who was coinfected with HIV, after 24 months of linezolid therapy. However, she was continued on linezolid at a reduced dosage and the dose of pyridoxine was increased. Her symptoms (burning sensation in soles of feet) subsided without any sensory deficit.

Rho PJ et al. reported an elderly patient with well controlled diabetes who underwent total knee arthroplasty and was subsequently treated for a chronic infection, who suffered peripheral neuropathy after 10 months of linezolid therapy. Features of neuropathy resolved after stopping linezolid [9]. Reversal of linezolid induced neuropathy after drug cessation has previously been reported in an adult treated for DRTB [10]. To the best of our knowledge, there have not been any case reports highlighting the occurrence of peripheral neuropathy in a child with diabetes on long-term treatment with linezolid.

Our patient had received linezolid for 8 months prior to developing features of peripheral neuropathy. When used for this duration, linezolid can act on mitochondrial DNA and affect protein synthesis. This can result in respiratory chain dysfunction. In animal experiments, linezolid induces a dose- and time-dependent decrease in the activity of respiratory chain complexes containing mtDNA-encoded subunits with a decreased amount of protein of these complexes. The amount of mtDNA remains normal [11]. Peripheral neuropathy usually begins in the lower limbs and is usually of sensory-motor axonal type. Common symptoms include pain, numbness, tingling, burning, and allodynia, usually occurring in a glove- and stocking-like distribution. While optic neuritis due to linezolid has been shown to be reversible in many instances, reversibility of peripheral neuropathy has been described as limited. In addition, our patient had a transient haematological abnormality (thrombocytopenia) not requiring intervention. As is evident from other case reports, this discordance of haematological and neurological abnormalities due to linezolid is well recognized [12–14]. Haematological and neurological side effects are less common in children than in adults. This difference may partly be accounted for by a reduced susceptibility of children to mitochondrial toxicity and a less frequent need for long-term treatments [15].

Our patient also received ethambutol due to evidence of susceptibility in the DST result; this drug has also been reported to cause peripheral neuropathy rarely [16]. Peripheral neuropathy has also been mentioned as a rare but possible adverse effect of two other drugs he received: cycloserine [17] and prothionamide [18].

In addition to the potential for adverse events, TB and DM can be a challenging combination to treat. DM can inhibit the host immune response to *M. tuberculosis* by decreasing T lymphocyte and neutrophil counts, decreasing Th1 cytokine response, and inhibiting macrophage function. In addition, TB, and some anti-TB drugs, can worsen glycaemic control, making complications like neuropathy more likely and diabetic management difficult [19, 20]. Enhanced glucose control is more effective at preventing neuropathy in patients with type 1 than in those with type 2 DM [21]. A well-coordinated multi-disciplinary approach is necessary to optimally manage the two conditions. The link between DM and TB and the implementation of the collaborative framework for care and control have the potential to stimulate and strengthen the scale-up of non-communicable disease care and prevention programmes, which may help in reducing not only the global burden of DM but also that of TB [22].

## Conclusion

The presence of both DM and TB should alert a physician to strive for optimal glycaemic control to minimize the risk of complications as well as optimizing the chances of recovery from TB. In addition, regular monitoring for the adverse effects of linezolid, especially in patients with DM and when the drug is used over a long period of time, is essential to enable early detection and appropriate management to avoid chronic painful neuropathy. In this case, the neuropathy improved with cessation of linezolid.

## Abbreviations

(XDR)-TB: Extensively drug-resistant
DM: Diabetes mellitus
DST: Drug susceptibility test
MDR: Multidrug-resistant
TB: Tuberculosis

## References

[1] WHO treatment guidelines for drug-resistant tuberculosis 2016 update. © World Health Organization 2016. Online http://who.int/tb/MDRTBguidelines2016.pdf?ua=1 27748093

[2] Riza AL, Pearson F (2014). Clinical management of concurrent diabetes and tuberculosis and the implications for patient services. Lancet Diabetes Endocrinology.

[3] Jeon CY, Murray MB: Diabetes mellitus increases the risk of active tuberculosis: a systematic review of 13 observational studies. PLoS Med. 2008, 5: e152–10.1371/journal.pmed.0050152 10.1371/journal.pmed.0050152 PMC2459204 18630984

[4] Singh R, Kishore L, Kaur N (2014). Diabetic peripheral neuropathy: current perspective and future directions. Pharmacol res.

[5] Wu Y-B, Li H-Q (2013). CHOP/ORP150 ratio in endoplasmic reticulum stress: a new mechanism for diabetic peripheral neuropathy. Cell Physiol Biochem.

[6] Agyeman AA, Ofori-Asenso R (2016). Efficacy and safety profile of linezolid in multi drug-resistant and extensively drug-resistant tuberculosis: a systematic review and meta analysis. Ann Clin Micobiol Antimicrob.

[7] Anger HA, Dworkin F et al. Linezolid use for treatment of multidrug-resistant tuberculosis and extensively drug-resistant tuberculosis, NewYork City, 2000–06. 10.1093/jac/dkq017 20150181

[8] Rose PC, Hallbauer UM (2012). Linezolid containing regimens for the treatment of drug resistant tuberculosis in south African children. Int J Tuberc Lung dis.

[9] Rho JP, Sia IG (2004). Linezolid associated peripheral neuropathy. Mayo Clin Proc.

[10] Vishnu VY, Modi M, Goyal M, Lal V (2016). Linezolid induced peripheral neuropathy. American J of Ther.

[11] De Vriese AS, Coster RV, Smet J (2006). Linezolid-induced inhibition of mitochondrial protein synthesis. Clin Infect dis.

[12] Bressler AM, Zimmer SM (2004). Peripheral neuropathy associated with prolonged use of linezolid. Lancet Infect dis.

[13] Park IN, Hong SB (2006). Efficacy and tolerability of daily half dose linezolid in patients with intractable multidrug-resistant tuberculosis. J Antimicrob Chemother.

[14] Nambiar S, Rellosa N et al. Linezolid induced peripheral and optic neuropathy in children. Pediatrics. 2011 Jun; 127 (6): e: 1528–32. 10.1542/peds.2010-2125 21555496

[15] Chiappini E, Conti C, Galli L (2010). Clinical efficacy and tolerability of linezolid in pediatric patients: a systematic review. Clin Ther.

[16] Nair VS, Lebrun M, Kass I (1980). Peripheral neuropathy associated with ethambutol. Chest.

[17] Varaine F, Rich ML. Tuberculosis: Practical guide for clinicians, nurses, laboratory technicians and medical auxiliaries. Medecins Sans Frontieres and Partners in Health; 2014 edition.

[18] Girling DJ (1982). Adverse effects of antituberculosis drugs. Drugs.

[19] Niazi AK, Kalra S (2012). Diabetes and tuberculosis: a review of the role of optimal glycemic control. Journal of Diabetes & Metabolic Disorders.

[20] Bodnar T, Starr K, Halter JB. Linezolid associated hypoglycemia in a 64 year old man with type 2 diabetes. Am J Geriatr Pharmacother 2011; Feb:9(1):88–92. 10.1016/j.amjopharm.2011.02.002 21459312

[21] Peltier A, Goutman SA, Callaghan BC (2014). Painful diabetic neuropathy. BMJ.

[22] Harries AD, Kumar AMV, Satyanarayana S, Lin Y, Zachariah R, Lonnroth K, Kapur A. Diabetes mellitus and tuberculosis: programmatic management issues. Int J Tuberc Lung Dis 19(8): 879–886. 10.5588/ijtld.15.0069 PMC4497633 26162352

